# Advance Directives in Oncology and Haematology: A Long Way to Go—A Narrative Review

**DOI:** 10.3390/jcm11051195

**Published:** 2022-02-23

**Authors:** Kevin Serey, Amélie Cambriel, Adrien Pollina-Bachellerie, Jean-Pierre Lotz, François Philippart

**Affiliations:** 1 Anesthesiology and Intensive Care Medicine Department, APHP—Ambroise Paré University Hospital, 92100 Boulogne-Billancourt, France; kevin.serey@aphp.fr; 2 REQUIEM (Research/Reflexion on End of Life Support Quality in Everyday Medical Practice) Study Group, 75015 Paris, France; amelie.cambriel@aphp.fr (A.C.); pollina-bachellerie.a@chu-toulouse.fr (A.P.-B.); jean-pierre.lotz@aphp.fr (J.-P.L.); 3 Anesthesiology and Intensive Care Medicine Department, APHP—Tenon University Hospital, 75020 Paris, France; 4 Anesthesiology and Intensive Care Medicine Department, Toulouse Hospitals, 31000 Toulouse, France; 5 Pôle Onco-Hématologie, Service D’oncologie Médicale et de Thérapie Cellulaire, APHP—Hôpitaux Universitaires de L’est Parisien, 75020 Paris, France; 6 Medical and Surgical Intensive Care Department, Groupe Hospitalier Paris Saint Joseph, 185 Rue R. Losserand, 75674 Paris, France

**Keywords:** end of life, advance directives, advance care planning, intensive care, medical oncology, malignant hemopathy

## Abstract

Patients living with cancer often experience serious adverse events due to their condition or its treatments. Those events may lead to a critical care unit admission or even result in death. One of the most important but challenging parts of care is to build a care plan according to the patient’s wishes, meeting their goals and values. Advance directives (ADs) allow everyone to give their preferences in advance regarding life sustaining treatments, continuation, and withdrawal or withholding of treatments in case one is not able to speak their mind anymore. While the absence of ADs is associated with a greater probability of receiving unwanted intensive care around the end of their life, their existence correlates with the respect of the patient’s desires and their greater satisfaction. Although progress has been made to promote ADs’ completion, they are still scarcely used among cancer patients in many countries. Several limitations to their acceptance and use can be detected. Efforts should be made to provide tailored solutions for the identified hindrances. This narrative review aims to depict the situation of ADs in the oncology context, and to highlight the future areas of improvement.

## 1. Introduction

Cancer is becoming more frequent as the population ages. An increase of 60% of the incidence between now and 2040 is foreseen [1,2,3]. Although great therapeutics improvements have been made, cancer remains a very severe condition. Both cancer and its treatments are responsible for patients’ weakening condition [4,5], thereby sowing the seeds for acute illnesses and potential need for intensive care [6,7]. The major issue in such context is to define the potential relevance of critical care. Although the prognosis of cancer patients requiring intensive care unit (ICU) has improved during the last decades [8], their mortality is very high [9,10], and residual morbidity is common among survivors [4,11]. Survival is associated with autonomy loss and psychological disorders [12], leading to severe impairments in patients’ short- and long-term quality of life [13].

When ICU admission is debatable, the patient’s opinion on life sustaining treatments, invasive care, possible alteration in autonomy and quality of life should be at the center of the decision-making process. Whether the expected benefits of intensive care are certain or not, this information should be obtained beforehand as patients should be given the possibility to accept the burden and possible consequences coming with invasive treatments.

Unfortunately, the severity of conditions requiring an ICU admission often goes along with consciousness’ alterations and usually prevents patients from expressing their preferences and wishes. In such circumstances, physicians turn to relatives to gather as much information as possible regarding the patient’s wishes. However, correlation with patients’ thoughts is surprisingly poor [14,15].

Lack of certainty about patients’ outcome and preferences leads to wide variability in the assessment of critical care relevance between physicians [16]. This uncertainty may lead to a standby resuscitation allowing time to gather these elements. However, this standby resuscitation may be invasive and/or induce inadequate ICU admissions when end-of-life care would have been deemed more appropriate by patients and relatives [17]. Moreover, inappropriate aggressive care, although sometimes necessary while waiting for complementary information, is associated with worse patient quality of life and a higher risk of major depressive disorder in bereaved relatives [18].

Patients’ preferences regarding care intensification and invasive treatments, as well as their beliefs, life- and end-of-life goals, perceived health-related quality of life, along with medical reasoning, may help the physician find the right balance between diagnostic or therapeutic procedures and patient comfort-oriented care.

In response to this imperative necessity, the concept of Advanced Directives or Advance Care Planning (ACP) in English speaking countries was developed in the middle of the 20th century in the USA by Luis Kutner [19]. Since 1976, similar notions have been introduced in many national legislations, following the Californian initial momentum. Advanced care plans can gather any indication one deems to be important for his care. Of note, depending on the country, ACP and AD do not mean exactly the same thing. They are usually distinguished by the temporality of their function. ACP may refer to the plan of chronic healthcare concerning either or not a specific chronic disease. On the other hand, the role of AD is often limited to the expression by the patient of anticipated care preference in response to hypothetical situations of acute and severe disease (or complication) were they were unable to speak for themselves. Nonetheless APC, by taking into account the philosophy of life of patient may contribute to physician emergency decision in absence of AD. While the absence of ACP is associated with a greater probability of receiving unwanted intensive care, their existence is associated with better respect of the patient’s desires and greater satisfaction regarding received care [20].

In several studies around the world, patients reported a strong interest for advance directives (AD) or ACP [21,22,23,24,25]. Nonetheless, the percentage of people who effectively have AD remains very low in the general population [26,27]. It scarcely exceeds 50% of oncology or hematology patients in some North American reports [28,29,30,31,32,33,34,35] and has been estimated to be as little as 5% in other [36]. 

**Figure 1:**
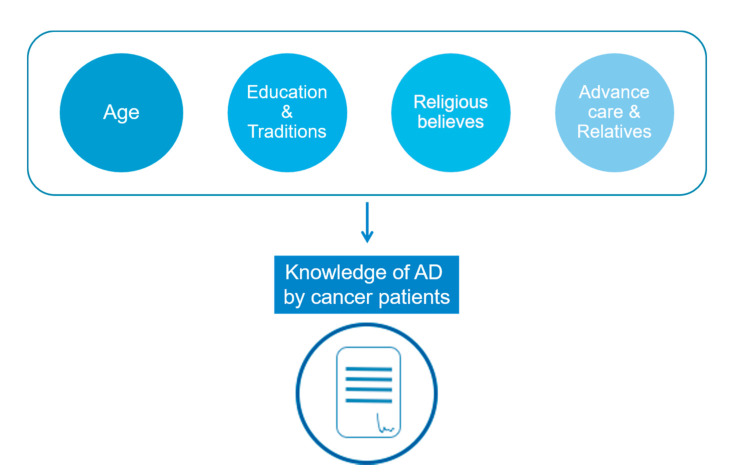
Main obstacles for advance directives information and use in cancer patients.

The aim of this review is to look at factors associated with the presence or absence of Advance Directives. We will look at factors depending on patients, the disease itself, physicians, as well as fears and misconceptions from both stakeholders (synthetized in Figure 1). 

## 2. Method

For this narrative review, we addressed the question of factor favouring or limiting advance directive use described in the literature. Studies were identified by a double search in PubMed/MEDLINE (National Library of Medicine) databases until December 2019 by two independents authors (KS and AC). The following keywords were used: (“advance care planning” or “advance directive”) AND AND “cancer” OR “cancer patient” OR “patient with cancer” OR “malignant tumor” OR “neoplasia” OR “malignant hemopathy” OR “hematological malignancy” OR “leukemia” OR “lymphoma”). Article relevance was refined by using the advance search builder tool of PubMed restricting to keywords presents in “title or abstract”. The final selection of paper was made by the authors, function of relevance to the addressed question. Additional articles, cited in the selected ones were included function when considered of major importance in the field.

## 3. Patients

**Figure 2:**
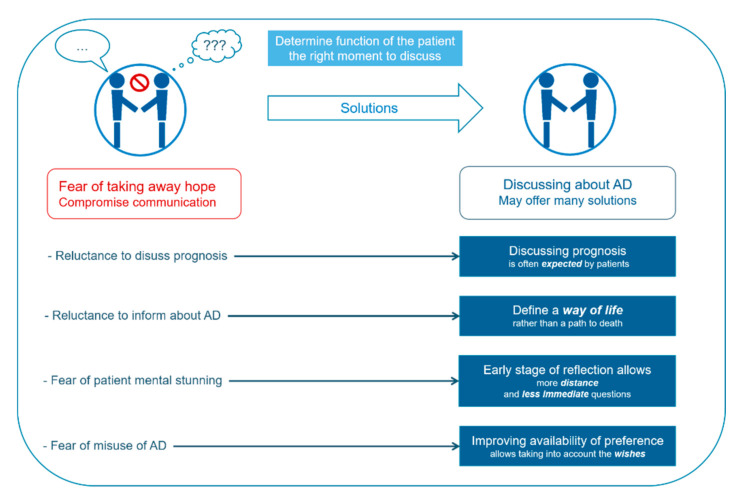
Main parameters associated with knowledge of advance directives.

Parameters associated with knowledge of AD by patients are synthetized in Figure 2.

### 3.1. Demographic Characteristics

In multiple occidental studies, older age seemed to be directly correlated with the redaction of AD [24,26,31,34,37,38]. It seems reasonable to suppose that older patients have a clearer idea of their end-of-life wishes and may be more prone to favour a good quality of life over a longer life. There is no other demographic parameter, such as gender, demonstrated to be linked to having AD [28,30,32].

### 3.2. Education and Social Characteristics

Socio-economic and educational background are highlighted as independent influencing factors in multiple works [37,38,39,40,41]. Both higher education and higher income are associated with the redaction of AD [28,42]. 

In a study conducted in an outpatient cancer clinic in Ohio, the authors suggested that people with low income and low educational background may have other concerns than discussing their preferences for treatment or end of life care, or that they may have less access to assistance or education programs on ACP [32,42]. However, a higher socio-economic status is not constantly brought up as a favouring factor in the literature. On the contrary, a Korean study from 2017, while also finding an association between age or higher education and AD’s redaction, revealed that participants with a low economic status were more willing to express themselves on end-of-life [43]. According to the authors, since people with a low economic status may feel more anxious about the future, AD could relieve part of those concerns.

The discordance between those different studies may be explained by the fact that the study populations come from very different cultures.

### 3.3. Religious Beliefs and Traditions

Religious beliefs may also impact AD. It is interesting to note that their influence varies depending on the countries and creeds [44].

In a Chinese study, having religious beliefs was associated with having AD [39], mostly because respondents considered that healthcare providers would not pay enough attention to their faith otherwise. Other studies found the same association between being Catholic, Jewish or Protestant, and AD’s completion [45,46]. Even when religion is not associated with AD [47], spirituality does seem to favour reflection on health preferences as indicated in the study of Karches et al., where religiosity was not associated with AD’s redaction, but with having designated a decision maker [48]. Interestingly, Maciejewski et al. found that positive religious coping (believing that God is going to help and support the patient through difficulties) was associated with less AD’s completion, and a higher frequency of intensive care at the end of life [49]. Conversely, negative religious coping (considering that God has abandoned the patient and that the disease may be a punishment) was associated with higher AD’s completion [49]. Thus, religion can affect in either direction patients’ behaviour towards AD, and more generally towards end-of-life goals of care (preference for early palliative care or aggressive treatments).

On the same note, a patient’s culture also plays an important role [50]. In Eastern Asian cultures, burdening others is a disrespectful act, and talking about death is thought to bring bad luck on the person, therefore preventing an adequate prognosis disclosure and the expression of anticipated wishes. In China, where ADs are perceived less favourably, the traditional family involvement in decisions on level of care leads to very few ADs being written among cancer patients [39,51]. Such family influence in end-of-life care decisions also happens in occidental cultures [38,40,52]. 

Nonetheless, end-of-life care discussions between patients and their family, with or without counselling by a health-care worker, could improve AD’s acceptance [43,51]. Furthermore, improvement in AD’s consideration is possible when vital prognosis is engaged, especially if patients refuse to undergo therapeutic intensification to favour comfort care [22].

### 3.4. AD to Relieve Relatives

Family plays a central role both during the process of writing AD and at the time of end-of-life decisions [53]. In an American study of 2008, 73% of Latinos discussed ACP with their family but only 37% with their doctor [40]. They argued that writing ACP relieves their loved ones from making difficult decisions. Indeed, in the absence of AD, guilt or regret may push families to choose life-sustaining treatments over palliative care, no matter how aggressive and ineffective those treatments would be. 

In a survey conducted in the USA, 63% of patients reported that completing AD avoided placing responsibility for end-of-life decision-making on their loved ones, even though all participants believed that their family or friend would be able to make adequate decisions by themselves [45]. 

### 3.5. Awareness of AD

Lack of awareness regarding AD by patients is obviously a major obstacle. It ranges from complete ignorance of their existence or their usefulness, to redaction difficulties due to a lack of knowledge regarding their practical use or how to implement them.

Six years after AD’s legal apparition, a French survey including 367 hospitalized patients revealed that only 34.8% of them were aware of the possibility to have some [21]. However, after proper information, 93% of the patients were in favour of AD, suggesting that better communication could increase AD’s drafting. The urge to solve this issue is underlined in McDonald’s study where cancer patients themselves reported the lack of knowledge to be the strongest barrier to writing AD [28]. In Hubert’s study, the need for more information was the reason why half the patients had not written their AD [34]. To raise awareness on AD and promote discussion with physicians or relatives, question prompt lists on end-of-life care seem to be efficient [54,55].

People may also have trouble figuring out how their AD can specifically relate to their disease. In a survey conducted in Germany in 2014, half of the patients were in favour of AD consultations. However, only 20% reported that their interest for those consultations was associated with their cancer diagnosis whereas the link between a severe chronic disease and the benefit of expressing themselves about their preferences concerning therapeutic intervention in case of acute and critical situation remains seems not to be obvious [56]. This work underlines the necessity to properly inform patients and physicians on the role and use of AD, and why a new serious disease may be a good time to think about one’s wishes for his care.

## 4. Sickness

Suffering from a deadly disease compels patients to think about their end of life. Unsurprisingly, a new diagnosis of cancer and duration of the illness turn out to be favouring factors towards AD’s writing [22,30,56].

### 4.1. The Importance of Prognosis

Both the ongoing disease and the patient’s past history play a role in the perception of AD. Sahm et al. noted in 2005 that patients having a previous experience of serious illness increasingly reported their intention to write AD [23]. In the same cohort, patients who often experienced pain as well as those deeming to be in a bad state of health state also reported more frequently their intention to draft AD [23].

Realizing the severity of one’s condition seems to be a trigger to AD’s drafting [51]. A longer self-assessed life-expectancy is associated with a lower likelihood of do-not-resuscitate order and a higher preference for life-prolonging over comfort-oriented care [57]. In a study including people without written AD, the proportion of patients in favour of a specific consultation on AD doubled between those with a non-malignant disease and those with a malignant disease and a life expectancy shorter than 6 months [56]. Similar observations were made with curative treatments: patients who wrongly overestimated their survival rate were far more likely to favour life sustaining therapies care [58,59].

However, even when prognosis is discussed with patients, it may not be well understood. A multicentre study including nearly six hundred metastatic cancer patients, revealed that even when informed of their prognosis by their physician, nearly a third of patients overestimated their life expectancy by more than 2 years. Correct recall of prognostic disclosure was associated with a more realistic assessment of their life expectancy [57].

### 4.2. Mental Stunning

When information about the disease, its treatment and its prognosis is delivered during the cancer clinic visit, patients may be stunned, impairing their ability to process and understand those details [60]. Some may be concerned about the psychological repercussions for the patient of the announcement of a pejorative prognosis right after being informed of their diagnosis. However, George et al. found no lasting psychological harm amongst terminally ill patients after they understood their prognosis [61]. Addressing these psychological factors may help patients better understand the ins and outs of their care and therefore choose the most appropriate care [60].

### 4.3. Link between End-of-Life Care and Anticipated Discussion

Encouraging early communication between physicians and patients regarding the prognosis of the disease could help patients to better prepare for possible complications or tragic outcomes, and to refine their goal of care [62]. Many papers showed that patients who discussed their end-of-life preferences early in the history of their illness with their physicians were more likely to report a greater well-being, and to receive fewer aggressive interventions in their last weeks of life, without survival time reduction [63,64,65].

Ganti et al. retrospectively explored the relation between engagement in ACP amongst patients receiving a hematopoietic stem-cell transplantation (HSCT) and adverse outcomes, as well as overall survival. They found that patients who engaged in ACP before HSCT had a better one-year and overall survival compared with patients without ACP [30]. Though no direct causal relation could be suspected, it is possible that those who did not engage in ACP were less prepared to face complications when they occur.

## 5. Healthcare Workers: Reluctance to Provide Information on AD

As previously addressed, the absence of AD in the cancer patient population is partly due to the absence of information on this legal disposition. This lack of knowledge is partly due to the reluctance of clinicians to discuss patients’ wishes [66].

In a study analyzing discussions during medical appointments between advanced cancer patients and their oncologists, treatment was mentioned in 94.3% of visits whereas prognosis was only disclosed in 50.4% of them [67]. Lower percentages of prognosis disclosure are underlined in other studies [68]. In extreme cases, this delay could lead to an absence of information about the severity of the prognosis until it is too late [69].

### 5.1. Reluctance to Discuss Prognosis

Many physicians feel inappropriately trained or prepared to talk about AD [66,70]. However, younger physicians are more prone to discuss AD sooner in the care [71,72]. Oncologists often feel responsible to initiate discussions about prognosis, but they believe that patients have to give them clues about what they are ready to hear [73]. While the absence of questions from patients may be interpreted as a wish to remain in the dark regarding their prognosis, [74], many patients expect physicians to raise the subject of end of life and ACP [23,73,75] and figure that their doctor is not at ease with the matter since the subject is avoided [75]. To address the issue, Freedman offered an approach respecting the wish of patients to know or to not know: he suggested that physicians should “offer truth” and then respect the patient’s choice [76].

### 5.2. Fear of Taking Away Hope

Qualitative studies report that oncologists feel that discussing end-of-life issues is emotionally difficult both for them and patients [66]. Indeed, patients seek hope and reassurance from their physicians as they “go to an oncologist to be cured, not to be buried” [77]. Physicians may postpone raising the subject as they sometimes fear that talking about death will be perceived by the patient as an indirect message that he is dying, thus triggering worries and fears [67] and possibly deteriorating the doctor-patient relationship [78]. However, focusing only on treatments may jeopardize opening discussion about end-of-life issues [66].

Several studies suggest that it is possible to talk about end-of-life preferences and write AD without interfering with the patient’s hope [54,79,80,81]. AD can even help terminally ill patients to reach a sense of control and peace of mind [81]. Moreover, since prognosis is almost impossible to determine with certainty at the time of diagnosis and may improve over time thanks to new treatments and study protocols [82], prognosis estimations should be refined over time, therefore initially authorizing the patient for hope [83].

### 5.3. Finding the Right Timing

Despite a general agreement on the necessity of AD [41], the best timing to write them is a matter of debate. Some physicians report they are uncomfortable treating and implementing palliative care for a patient at the same time, as it may seem contradictory [66]. They think that the best timing to start a discussion on end-of-life issues and AD is when the patient becomes terminally ill [41,71,78].

Delaying the discussion may not ease the process: when there is no curative option left, the change in goal of care is even more abrupt and causes more distress [84]. Postponing ACP discussions until patients seem ready or ask for it is probably not the best alternative either, as a large part of the patients (between 30 and nearly 50%) will wait, preferring their doctor to break the subject [75,85,86].

Patients themselves reported that AD completion or dedicated consultations should take place early in the illness [41]. Indeed, early discussion of goals of care probably remains the best way to provide a person-centered care throughout follow-ups. Waiting for patients’ acceptance while periodically reminding them to think about AD could be part of the solution [87,88].

### 5.4. Lack of Time

Lack of time during oncology consultations is an identified barrier to end-of-life care discussion and AD’s completion [56,67]. In a British study, one third of patients reported that they lacked time during clinic appointments to discuss AD [75].

Distilling prognosis and end-of-life information throughout the follow-up may be less time-consuming, could favor discussion, and may strengthen the patient–physician relationship, therefore giving patients a better chance to express their desires regarding their care [84].

## 6. Fear of Misuse

Some obstacles to the realization of AD are due to physicians’ and patients’ misbeliefs or fears [89]. Some patients think that AD are dangerous because they may become inadequate in an unplanned acute situation [90]. They fear that AD could dictate the physicians’ role, thus leading to less invasive care regardless of the context [23,90]. This underlines a lack of information on AD in three ways: practical use of this tool, possibility to modify AD at any time, as well as their integration in the healthcare reflection.

Other patients worry that their wishes may not be respected [23], as it was underlined for Do Not Resuscitate (DNR) orders [91]. However, the first reason for AD transgression may just be the ignorance of their existence [91]. In the USA, the Physician Orders for Life-Sustaining Treatment (POLST) program recommends to always keep a copy of the patient’s form that should follow him in case of a hospital transfer for example [92]. However, this is not a fool-proof method either, and the lack of interoperability between electronic health records in various facilities is a problem still to resolve [93].

Another barrier to AD is the fear of coercion. Sahm et al. explored this concern amongst patients, healthy controls, nursing staff and physicians [23]. They found that more than half of the participants in all groups worried that patients could be pressured to write AD. However, although AD remains optional, it is important to keep in mind that many patients also identify AD as a facilitator to discuss end of life care [38] and that they have proven to foster tailored care and to improve patients’ quality of end-of-life [94].

## 7. Discussion: How to Improve AD’s Generalization?

ADs are effective tools to improve the quality of terminally ill patients’ end-of-life. In a systematic review, Arianne Brinckman-Stoppelenburg highlighted an improvement in quality of life for both patients and their family, a higher compliance with patients’ preferences, and a decreased use of aggressive care at the benefit of palliative care when AD were redacted [94].

### 7.1. AD for Who?

Overall, despites multiple attempts to identify favourable or unfavourable factors to the completion of AD, it is difficult to recognize patients who would most benefit from AD’s drafting.

First, we ought to understand that patients may not want to choose in advance which treatments they will seek or reject in the future, and that they may prefer to rely on their relatives or caregivers. This ultimate expression of autonomy is summarized by Megan-Jane Johnstone as “the choice to not have to choose” [95].

As stated by Stephanie Johnson, “individual autonomy is socially dependent” (page 384) [96]. The priority is to concentrate on the meaning each patient gives to their illness, on their preferences for their life and end-of-life. AD should not be just a mandatory list of dos and don’ts kept in medical records.

If patients are strongly opposed to writing AD, both having named a health care surrogate and having previously collected information about their wishes are valuable. Discussing AD does not necessarily mean writing AD, but it launches the reflexion process and favours the discussions.

### 7.2. When to Discuss about AD

The vast majority of patients seem to hope that such a topic will be discussed by the physician rather than initiated by them [23,71,78,96]. A recent study carried out in the United States highlighted the feasibility of more systematic information about AD during the initial management of patients suffering from severe tumour diseases (lung and digestive cancers, stage IV at management) [29]. This was recently confirmed by a study which again looked at information for patients with advanced tumour diseases (stage III and IV) at an early stage [97]. However, the ideal timing of information and of the request to write ADs differs from patient to patient and seems to depend on the prognosis of the chronic disease in the short and medium term. Nevertheless, it remains impossible to distinguish a homogeneous framework of preference according to chronic severity [56]. In a North American study, the patients with a severe disease were more likely to accept the discussion [56]. A similar distribution was observed in an Asian work (Korea), where the definition of the most relevant time of writing is variably defined according to the respondents (in the absence of significant pathology for about 17%, at the diagnosis of tumour disease for 30% and in the terminal phases of the disease for about 50% of patients) [41]. One time does not fit all, and the most important factor remains to initiate the discussion and the reflection about AD.

### 7.3. Discussing AD: With Who?

In an American study amongst cancer patients, 77% had discussed AD with their relatives and only 41% with a physician [24]. Moreover, only 22% of those who discussed them with a physician did so with their treating oncologist [24]. Besides, among those who had not previously discussed AD with their oncologist, only 23% reported they would like to do so. It seemed inconsistent for many of them to talk about death, palliative care, and discontinuation of treatment while being actively treated by the same oncologist [24]. Ironically, almost all patients were in favour of a policy in which patients would discuss AD with their admitting physician, even if they never met him before [24].

In many studies, patients would rather talk about AD with their general practitioner [24,34,78]. In a certain way it makes sense as it is usually the physician who knows the patient best [24]. However, the primary care physician may not always be the best interlocutor to help the patient face their specific malignant disease and possible complications. Yet, multiplying the range of health care correspondents allows for different perspectives on the subject, and also improves AD’s completion [33,98].

As previously mentioned, another pragmatic issue is the available time during oncological appointments. Thus, having other opportunities to discuss their goal of care may enhance patients’ participation in AD. Trained nurses can sensitize patients, either through question prompt lists or during non-scripted interviews they can handle in dedicated consultation [29,88]. Clinicians themselves considered it acceptable for all health workers to engage in goal of care discussions [71,99,100]. Having an advanced practice provider specifically initiate AD discussions seems to be effective [101].

### 7.4. What to Discuss and How to Write?

It is easy to realize how complex it could be for a patient to write their AD: they ignore what specific situation they will have to face, or how their document will be construed. Adding to the complexity of the exercise, patients can have difficulties understanding limitations and consequences of life-sustaining therapies [99].

The different information and wishes expressed by patients will probably not fit a restrictive questionnaire: a standardized form may be impersonal thus inappropriate. In a study involving general practitioners, they underlined how AD is about life and death and figuring out the patient’s wants and needs, and “that can’t be too calculated or tick boxed” (page e452) [78].

Contrary to DNR orders, AD should allow a great freedom of thought. There, patients can express all the complexity of the personal, material, spiritual and social aspects of their plan of care. Moreover, AD still enable the expression of hope while preparing for the end-of-life, whereas DNR are deemed “blunt” or “without any empathy” by patients [102]. It is then important to leave blank spaces in AD forms for unrestricted expression of the patient’s wishes.

## 8. Conclusions

AD and, more broadly, ACP are arrangements that allow patients to express their preferences regarding their therapeutic management, particularly in situations of chronic pathology where acute deterioration is always possible. Beyond choices regarding care, organ support or specific procedures, these arrangements can define the patient’s life values. More than just a written record of the specific elements that the person is likely to accept or refuse, AD and ACP should be a tool for reflection and an opportunity for a physician–patient exchange regarding the patient’s philosophy in order to improve the personalization of care. In spite of the steps taken, the use of AD remains very insufficient, especially in Europe, and this fact should encourage us to continue our efforts to both increase their application and to extend their scope of use.

## Data Availability

Not applicable.
